# Reflections on community mental healthcare around the world

**DOI:** 10.1192/bji.2020.12

**Published:** 2020-05

**Authors:** David Skuse

**Affiliations:** Professor of Behavioural and Brain Sciences, Division of Population, Policy and Practice, UCL Great Ormond Street Institute of Child Health, UK. Email: d.skuse@ucl.ac.uk

**Keywords:** Greece, Malawi, Cambodia, deinstitutionalisation, asylums

## Abstract

This editorial introduces three papers in *BJPsych International* on community mental healthcare in the diverse cultures of Greece, Cambodia and Malawi.

In this issue we begin with reflections on the provision of community mental healthcare in three diverse cultures. First, in Greece, where following the disestablishment of in-patient asylums little progress has been made in building an alternative model of care. Second, in Cambodia, where the entire mental health system was destroyed in the 1970s by the Khmer Rouge, and services have had to be redeveloped from scratch. Third, in Malawi, where the community already provides care through the medium of traditional healers but where the management of serious mental illness requires better integration with psychiatric expertise.

## Greece

The management of people with severe mental illness by incarceration in public asylums was the norm for many Western societies until relatively recently and was adopted by countries elsewhere in the world too, if they had been colonised by a power such as Britain or France.^1^ Greece came under the influence of the British, through their occupation of the Ionian islands in the early 19th century. The country's first psychiatric hospital was built in Corfu, although later mental health legislation was influenced by the French. Michael Madianos writes about psychiatric reform in Greece over the period 1999–2019.^2^ He emphasises that a mental healthcare system that was originally established nearly a century ago has changed relatively little, despite the considerable political developments that Greece has experienced in recent decades. Deinstitutionalisation, and the establishment of community care for mental disorders, began recently compared with Northern European countries, and reform has been slow to take root.

## Cambodia

In Cambodia, which gained its independence from France in 1953, there was a single psychiatric hospital, containing 2000 patients, at the beginning of the Khmer Rouge period during which, as Sarah Parry and Ewan Wilkinson describe, mental health services were totally destroyed.^3^ Rebuilding those services did not begin until the 1990s. The authors review progress in their article. Unfortunately, despite generous international funding soon after the Paris Peace Agreement was signed in 1991, this source of support has diminished in magnitude. There is a wish to develop community care for the many who have no access to services, but implementation of the strategic goals of the Cambodian government is still some way off.

## Malawi

Philippa Lilford describes, in vivid terms, her experiences as a junior psychiatrist in Malawi, which has but one mental hospital, in Zomba.^4^ The asylum's origins lie in the local prison, where it was established in 1910; it has existed in its current form since 1953. Criticism of the in-patient system, in the context of Malawian society, was made by a senior government psychiatrist at the hospital nearly 30 years ago.^5^ Dr Lilford spent some time there, and in another psychiatric facility that is attached to a general hospital. She reflects on the relationship between in-patient care and the local community, which still looks to traditional healers for support and guidance as a first resort.

All three papers have important points to make about the relationship between asylum and community care, and all three provide stimulating reading.
